# Objective Assessment of a Hearing Protection Device for Canines

**DOI:** 10.3390/vetsci13060564

**Published:** 2026-06-07

**Authors:** Joshuah B. Klutzke, George E. Moore, Emily S. Curry, Stephanie A. Thomovsky

**Affiliations:** College of Veterinary Medicine, Purdue University, West Lafayette, IN 47907, USA; jbklutzk@purdue.edu (J.B.K.); gemoore@purdue.edu (G.E.M.); escurry@purdue.edu (E.S.C.)

**Keywords:** canine hearing protection device, hearing threshold, brainstem auditory evoked response, noise-induced hearing loss

## Abstract

Noise-induced hearing loss (NIHL) has been reported in both people and canines and can raise the hearing threshold. In people, hearing protection devices (HPD’s) are utilized to reduce the risk of hearing loss. In canines, HPD’s have been developed, but none, to our knowledge, have been objectively tested to determine their ability to reduce incoming sound. We hypothesize that a canine hearing protection device (CHPD) will effectively protect hearing in canines by increasing hearing thresholds. Twenty-one apparently healthy canines between the ages of 4–69 months were anesthetized for routine surgical sterilization. Hearing thresholds were established using over-the-ear headphones (OTEH); starting at 70 dB nHL, sound was gradually diminished by 10 dB nHL until wave V was inapparent as recorded by BAER. Hearing thresholds were then established in the same patients while wearing CHPD. Median hearing thresholds for canines without the CHPD were 40 dB (range 5–70 dB) in the left ear and 50 dB (range 20–70 dB) in the right ear. Median hearing thresholds for canines with the CHPD were >70 dB AU (range 65–70 dB). Twenty out of twenty-one canines had hearing thresholds > 70 dB with the CHPD in place. The CHPD objectively increased the hearing threshold in canines, blocking sound of less than or equal to 70 dB nHL. This device could be utilized to lessen the likelihood of NIHL in canines.

## Simple Summary

Noise-induced hearing loss is a known complication in people who are exposed to loud noises for prolonged periods of time (i.e., in a workplace). These people are oftentimes given hearing protection devices while working to reduce the risk of hearing loss. Similar noise-induced hearing loss has been seen in dogs, but no hearing protection device has been objectively tested. In our study, we found a canine hearing protection device that can objectively reduce the amount of sound entering a dog’s ear. Use of this canine hearing protection device in settings where dogs are chronically exposed to loud noises (i.e., military/police dogs) may lead to a reduced chance of developing noise-induced hearing loss.

## 1. Introduction

Noise-induced hearing loss (NIHL) is the second most common cause of hearing loss in people and is estimated to affect at least 5% of the global population of people [1]. Cumulative hearing loss and immediate hearing loss can occur with sustained noise above 70 decibels (dB) or an instantaneous noise over 120 dB, respectively [1]. The hearing threshold, defined as the lowest intensity at which a sound can be heard, is a direct measure of hearing loss [1]. In the United States, regulatory bodies (specifically the Occupational Safety and Health Administration or OSHA) require employers to offer hearing protection to employees who are exposed to certain loud-noise situations in an attempt to lessen NIHL [2].

NIHL is likely underrecognized in canines, notably in working canines such as military, police, and hunting canines. These canines are more likely to be exposed to either instantaneous or prolonged loud noises [3]. Loss of hearing for these canines could lead to an inability to hear a handler’s commands, which may put the canine or handler in undue danger. Hearing loss is a significant concern for companion pet owners, as well. Owner perceptions of pet canine quality of life are negatively affected by hearing loss. Hearing loss has also been associated with behavior conditions in canines; there is the suggestion that hearing loss is a cause of anxiety-related disorders in canines [4,5]. While hearing protection devices have been developed for use in canines, to the authors’ knowledge, none have been objectively tested to determine if they affect hearing thresholds.

The primary aim of this study is to utilize brainstem auditory evoked response (BAER) testing to determine if a hearing protection device (CHPD) designed specifically for canines can increase the hearing threshold, effectively protecting the hearing of canines exposed to 70 dB or less of sound. We hypothesize the CHPD will effectively protect hearing in canines by increasing their hearing thresholds.

## 2. Materials and Methods

Shelter-owned canines presenting to the Purdue University Veterinary Hospital for elective ovariohysterectomy (OHE) or castration were enrolled in this prospective hearing study. Canines were recruited from January 2024 to April 2024. A complete physical examination was performed on each canine by a veterinarian (EC) at the time of study enrollment. Study consent forms were submitted to each shelter prior to enrollment. Study protocols were reviewed and approved by the university’s institutional animal care and use committee. Canines meeting the following criteria were included in the study: (1) aged between 4 months and 10 years old, and (2) weight between 10 kg and 30 kg. Canines were excluded if they had absent waveforms as recorded by BAER at 70 dB (using tubal inserts and/or OTEH) without CHPD in place and/or if they were determined on the physical exam to be unfit for general anesthesia.

Enrolled canines were pre-medicated with acepromazine 0.05 mg/kg and then anesthesia was induced utilizing a solution of tiletamine and zolazepam, butorphanol, and dexmedetomidine (TTDEX). The concentrations of each anesthetic agent within the solution include: tiletamine and zolazepam (Telazol, Zoetis Inc., Kalamazoo, MI, USA), 50 mg/mL and 50 mg/mL respectively; and butorphanol (Torbugesic, Zoetis Inc., Kalamazoo, MI, USA) 10 mg/mL, and dexmedetomidine (Dexdomitor, Zoetis Inc., Kalamazoo, MI, USA) 0.5 mg/mL. The agents were mixed as a solution at 5 mL of Telazol, 2.5 mL of butorphanol, and 2.5 mL of dexmedetomidine. The solution was administered at a dose of 0.03 mL/kg. Canines were then intubated and placed on isoflurane (Fluriso, MWI Animal Health Boise, ID, USA) gas for maintenance anesthesia and were placed in sternal recumbency; blood pressure, oxygen saturation (SpO_2_), and ECG were monitored continuously throughout the procedure.

### 2.1. BAER Protocol

**Figure 1:**
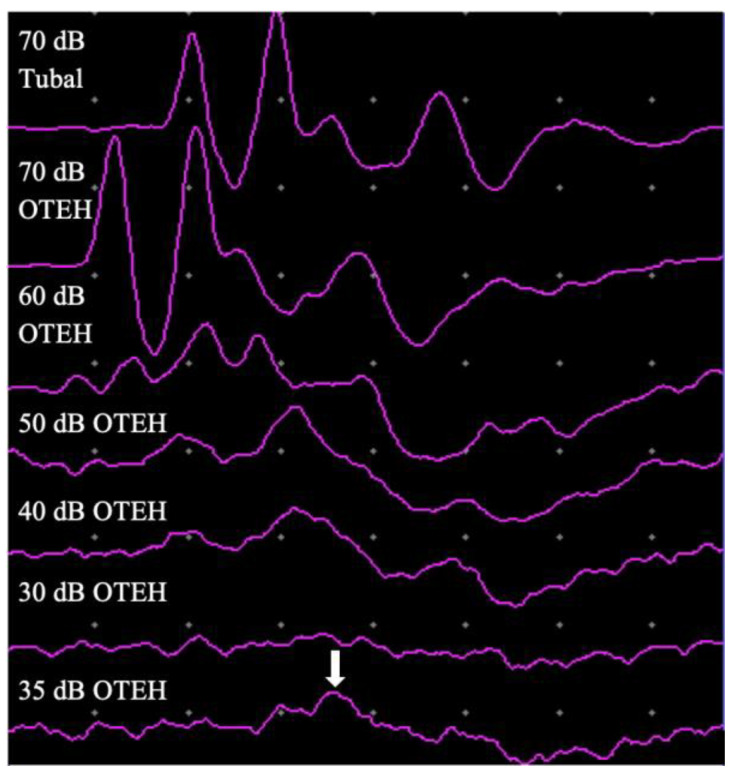
Example of BAER with decrements of dB showing progressive loss of waveforms. The loss of wave V is evident at 30 dB and the wave becomes visible again at 35 dB (white arrow), thus establishing the hearing threshold to be 35 dB.

**Figure 2:**
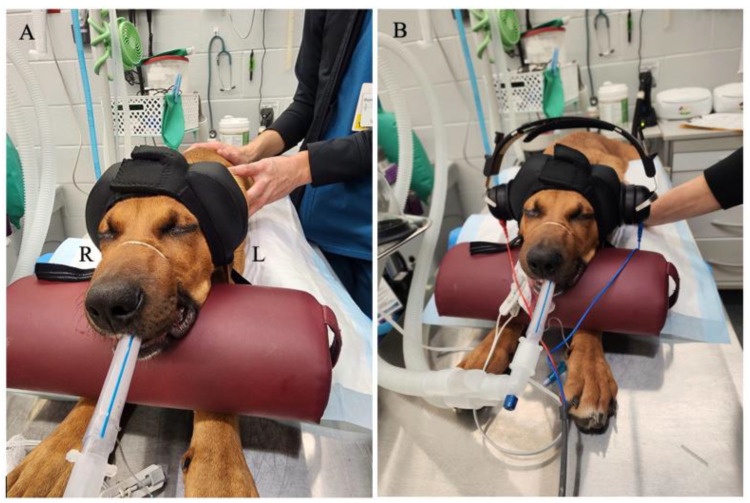
(**A**) Patient wearing the CHPD while under general anesthesia. (**B**) Patient wearing the CHPD with the OTEH in place.

A routine BAER with the T1 reference was performed. The patient had four subdermal electrodes placed—the ground electrode was placed on the nuchal crest, the reference electrode was placed over the T1 dorsal spinous process, and the recording electrodes were placed at the tragus of each ear being tested [6]. A 2-channel amplifier (Sierra Summit; Cadwell Industries Inc., Kennewick, WA, USA. Software used from https://www.cadwell.com) was utilized to perform the BAER. BAER set-up was as follows: stimuli: clicks; polarity: alternating; sweeps: 1000; and rejection: off. Due to the author having more experience using tubal inserts, hearing was first established (the presence of a normal BAER) at 70 dB normal Hearing Level (nHL) in both ears using tubal inserts. Hearing was determined to be intact if wave V was present [7]. Once hearing was established using tubal inserts, BAER was then recorded at the same dB level using over-the-ear BAER headphones (OTEH) (Cadwell Industries Inc., Kennewick, WA, USA). Both tubal inserts and OTEH were utilized to ensure accuracy of hearing threshold data prior to CHPD (Dawgmuffs LLC Justiceburg, TX, USA) placement. The hearing threshold was then established in both ears via OTEH by reducing the sound level by increments of 10 dB nHL (i.e 70 dB nHL, then 60 dB nHL, then 50 dB nHL, etc.) until wave V was no longer visible [7]. Following the loss of wave V, the dB nHL was increased by 5 dB nHL. The lowest dB value at which wave V was visible was recorded as the hearing threshold (Figure 1). Seventy dB nHL was the maximum tested as a safety measure for enrolled canines. The canine’s head was then measured and the appropriately sized CHPD (as per manufacturer’s recommendations) was securely placed on the canine’s head so that both ears were covered. The OTEH were placed on top of the CHPD (Figure 2), and the BAER was repeated to determine hearing threshold with CHPD using the same technique as described above.

Waveform latency was defined as the measurement between the beginning of the stimulus to the positive peak of each of the waves being measured [8]. This data was collected for all canines at each dB nHL of sound tested, and for both tubal inserts and OTEH. Waveform latencies were then calculated [8]. The median latency of each waveform, for each dB nHL level, was calculated for both tubal inserts and OTEH.

### 2.2. Statistical Analysis

Numerical variables were assessed for normality using the Shapiro–Wilk test. Parametrically distributed data were summarized as mean ± SD; ordinal and non-parametrically distributed data were summarized as median and range. Statistical analysis was performed analyzing repeated measures of ordinal/non-parametric data with Wilcoxon’s signed rank test. Statistical significance was set at *p* < 0.05.

## 3. Results

Twenty-three canines were recruited. Two of these canines were excluded; one canine had signs of vestibular dysfunction (horizontal nystagmus) and a negative BAER. The other canine was deemed physically unfit for general anesthesia. Thus, 21 canines were included. The most common breed was mixed breed canine (MBD) (10/21, 47.6%), followed by American Staffordshire Terrier (6/21, 28.6%), German Shepherd Dog (2/21, 9.5%), Husky (2/21, 9.5%), and Border Collie (1/21, 4.8%). The mean +/− SD estimated age was 27 months +/− 14.5 months and the mean +/− SD weight was 19.9 kg +/− 6.8 kg.

Median hearing thresholds for canines were 40 dB nHL in the left ear (AS) (range 5–70 dB) and 50 dB nHL in the right ear (AD) (range 20–70 dB). Median hearing thresholds for canines, when wearing the CHPD, were greater than 70 dB nHL in both ears (AU) (range 65–70 dB) (*p* < 0.001 AU) and 20/21 canines had hearing thresholds greater than 70 dB with the CHPD in place (*p* < 0.001). See Table 1.

The mean +/− SD wave V latency at 70 dB with the tubal inserts and OTEH was 4.36 ms +/− 0.1 ms and 3.44 ms +/− 0.5 ms, respectively. The difference between these two latencies was 0.92 ms.

## 4. Discussion

In the present study, a canine hearing protection device was objectively tested and showed it could block incoming sound of at least 70 dB nHL. In people, OSHA requires any employer to monitor noise exposure levels, with the goal of identifying employees who may be exposed to sounds greater than or equal to 85 dB for longer than 8 working hours in a day. Employers must provide these at-risk employees a baseline audiogram, which is then rechecked 1 year later, and annually thereafter. If employees show signs of hearing loss, appropriate hearing protection must be provided by the employer [9]. While veterinary medicine, in the United States, does not have these strict guidelines in terms of protecting hearing, a recent publication showed prolonged exposure for canines in a kennel-setting led to negative changes in hearing thresholds [10]. These objective findings support that hearing loss due to prolonged exposure to loud noise is not a uniquely human problem, but can also affect canines. Working and military canines may be exposed to acute or chronic loud noises such as gunshots, helicopters, and sirens; hearing acuity is imperative for these canines to perform their jobs well and the use of a hearing protection device could be helpful in prolonging their careers [3].

While protecting canines against NIHL is the primary goal of the CHPD, other potential uses could be considered. A recent publication showed that loud noise can negatively affect the quality of sedation in canines with dexmedetomidine [11]. Additionally, a study in people showed that when utilizing hearing protection during an orthopedic surgical procedure the patients required a lower total dose of propofol for general anesthesia [12]. With that in mind, procedures which require canines to be under general anesthesia while exposed to loud noise, such as an MRI, may benefit from hearing protection. Three Tesla MRI scanners produce sounds greater than what is considered safe by the United States FDA and OSHA; as a result, people undergoing these procedures are routinely given MRI-compatible hearing protection [13]. Lastly, hearing protection may lead to lower levels of anxiety in canines. A publication has shown lower amounts of salivary cortisol in canines wearing hearing protection for an MRI [14].

Due to the study design, a unique set of data was provided regarding the use of OTEH and tubal inserts when performing the BAER in our population of canines. Previous literature has reported a 1 msec delay in wave latency when using tubal inserts compared to OTEH [8]. The delay is secondary to sound traveling through plastic tubing within the tubal insert before arriving at the tympanic membrane [7]. In the current study, there was a 0.92 ms increase in latency of wave V when comparing tubal inserts to OTEH. This is a consistent finding (approximately 1 ms) with the previously published literature and helps support our BAER tests were performed correctly [15].

For study purposes, and to reduce the chance of a false-negative BAER, we had to ensure the canine’s pinna was fully contained within the OTEH while also not covering the external ear canal. We did this by pulling the pinna caudally and away from the canal before placing the OTEH on the canine. While there is the possible risk of a false-negative BAER if the external ear canal was covered by the pinna, none of the canines had absent waveforms at 70 dB nHL. Anesthetized canines were used to ensure accurate placement of the CHPD and OTEH; awake canines tend to move around during BAER testing, which could have introduced artifact in the BAER waveforms. The BAER is not significantly affected by anesthesia [7]. Lastly, the choice of 70 dB nHL as the highest sound tested was to avoid any chance of introducing any potential hearing damage to the canines being tested.

One limitation of this study was the use of only clicks as stimulus. While click stimuli test high frequency sounds, tone bursts can be utilized to test low frequency sounds [7]. In this study clicks were solely utilized for two reasons: this stimulus is what is traditionally utilized in BAER testing in clinical veterinary neurology practice [8] and we wanted to limit the time patients were under anesthesia. Testing for sound at a different frequency would have added to the patient anesthesia time.

CHPDs were fitted on patients following pre-medication and induction of anesthesia. During study development CHPDs were fitted to dogs without the influence of sedation or anesthesia. Subjectively dogs tolerated fitting and placement of the CHPD and fitting was easy.

Future testing considerations for this CHPD could include feasibility testing on military or working canines to determine if the CHPD can be utilized while in the field; evaluating the effect the CHPD has on canine anxiety (i.e., in a hospital or boarding facility); and use of the CHPD to reduce the use of anesthetic drugs during a variety of diagnostic and surgical procedures.

## 5. Conclusions

The CHPD did objectively increase the hearing threshold in healthy canines, blocking sound of less than or equal to 70 dB nHL and could be utilized to lessen the likelihood of NIHL in canines.

## Figures and Tables

**Table 1:** Median and range of hearing thresholds for the left ear (AS) and the right ear (AD) with and without the CHPD in place.

	AS Without CHPD	AD Without CHPD	Wearing CHPD AS	Wearing CHPD AD
Hearing thresholds (dB)—median (range)	40 (5–70)	50 (20–70)	70 (70–70)	70 (65–70)

## Data Availability

The original contributions presented in this study are included in the article. Further inquiries can be directed to the corresponding author.

## Abbreviations

The following abbreviations are used in this manuscript:

NIHL	Noise-Induced Hearing Loss
HPD	Hearing Protection Device
CHPD	Canine Hearing Protection Device
OTEH	Over-The-Ear Headphones
dB	Decibels
BAER	Brainstem Auditory Evoked Response
OHE	Ovariohysterectomy
nHL	Normal Hearing Level
